# Peer Review in a Post-Eprints World: A Proposal

**DOI:** 10.2196/jmir.2.3.e14

**Published:** 2000-09-01

**Authors:** James Till

**Affiliations:** ^1^Joint Centre for Bioethics and Department of Medical Biophysics, University of Toronto and Division of Epidemiology, Statistics and Behavioural Research, Ontario Cancer InstituteCanada

**Keywords:** Internet, Computer Communication, Electronic publishing, Cybermetrics

## Abstract

Recently, a number of electronic biomedical preprints servers, which allow the archiving of electronic papers without prior peer review, have been established, most notably the Clinical Medicine & Health Research NetPrints website and the The Lancet's Electronic Research Archive. These mark an extension to clinical medicine and health research of a novel experiment in the provision of public access to electronic versions of preprints. However, until now the biomedical community has been slow to adopt this new form of communication. This paper discusses how the value and attractiveness of eprint servers can be improved, and how electronic preprints (eprints, NetPrints) can be evaluated. Previous studies of variations in rejection rates after conventional peer review have indicated that the extent of scholarly consensus is an important variable for acceptance. This variable seems likely also to be important in readers' and editors' evaluations of eprints. A combination of unsolicited comments together with commissioned review might yield articles of higher quality than either could accomplish alone. However, if systematically applied to all eprints, such a process would be time-consuming and labor-intensive. A sequential review process is proposed, beginning with the acceptance of a preprint by an eprint server, followed by revision on the basis of comments received publicly or privately, and by the solicitation of selected eprints for commissioned review. This sequential process could have advantages, both for the authors of articles, and for journal editors. For example, the eprint would, in effect, have been submitted simultaneously to a large number of relevant journals. Some issues about evaluative studies of the outcomes of eprint submissions are also considered briefly. It would be particularly valuable if every eprint server included access to comparative statistics on visits by readers to individual eprints.

## Background

The establishment of BMJ's Clinical Medicine NetPrints [1] and The Lancet's Electronic Research Archive [2] websites marks an extension to clinical medicine of a novel experiment in scientific publishing. The experiment involves public access to electronic preprints, without prior peer review. The arXiv archives [3], now involving preprints in physics, mathematics, nonlinear sciences and computer science, are probably the best-known, but other archives are participating (for example) in the Open Archives Initiative [4]. Although the term "eprints" is a generic one that could be applied either to electronic preprints ("e-preprints") or to electronic reprints ("e-reprints," or "e-postprints"), this article will be mainly about electronic preprints. These are referred to as "NetPrints" at the BMJ's website [1], and as "Eprints" at The Lancet's website [2]. Facilities for the storage and dissemination of electronic preprints will be referred to here as "eprint servers" or "eprint archives."

There has been much controversy about proposals to extend to the biomedical sciences a concept first adopted by a sub-group of physical scientists. To what extent is it appropriate to apply, more widely, experience "drawn from a well-defined and highly interactive community of voracious readers with a pre-existing hard-copy preprint habit ..." [5]?

A proposal designed to foster electronic publications in the biomedical sciences (originally called "E-Biomed," but subsequently modified and renamed "PubMed Central" [6]), was strongly criticized, especially by editors of The Lancet [7], and the New England Journal of Medicine [8]. It was pointed out that, in basic research, scientists are communicating primarily with one another, and (in comparison with clinical research) the immediate practical consequences of a mistake are not as great and are easily corrected [8].

On the other hand, a major issue addressed by these websites is the information and communication needs of researchers and health professionals in resource-poor countries [2]. The concern that inadequately-evaluated eprints with significant public-health implications might cause unnecessary harm is addressed via editorial checks prior to posting at The Lancet's website [2], and via an explicit warning at the ClinMed NetPrints website [1] that the eprint has not yet been peer-reviewed. The latter website, which more closely resembles the arXiv archives [3], will be the main focus of the present article.

Stated purposes of the ClinMed NetPrints website include provision of access to electronic preprints of articles, and access to facilities for direct reader feedback prior to eventual publication in a paper journal [9]. In an editorial announcing the BMJ's website [9], it is stated that: "We have always regarded publication in the paper journal as not the end but rather only part of the peer review process. Every editor has seen published studies destroyed in the correspondence columns."

It is increasingly widely accepted that the conventional peer review of manuscripts is "expensive, slow, prone to bias, open to abuse, possibly anti-innovatory, and unable to detect fraud," and can yield published papers that "are often grossly deficient" [10]. A publication process in which correspondence columns are used to "sort out the good from the bad and point out the strengths and weaknesses of studies" [10] has not been compared with conventional peer review. And, "most studies have compared one method of peer review with another and used the quality of the review as an outcome measure rather than the quality of the paper" [10].

The remainder of the present article is divided into four sections. In the first, a problem (variable rejection rates) that might be expected to differ for eprints in comparison with conventional peer review is considered. In the second, a case study of a "gold standard" for electronic journals, involving a combination of online peer review with a second appraisal process (online comments from readers) is reviewed. In the third, a proposal about ClinMed NetPrints, involving a sequential process, initially providing an opportunity for readers to comment, followed by an invitation for selected NetPrints to be submitted for conventional peer review, is outlined. Finally, in a concluding section, some issues about evaluative studies of eprints are outlined briefly.

## Variable Rejection Rates after Peer Review: Role of "Scholarly Consensus"

In 1971, Zuckerman and Merton [11] published an article about variation in rejection rates across journals in different disciplines. They reported substantial variation, with rejection rates of 20 to 40 percent in the physical sciences, and 70 to 90 percent in the social sciences and humanities. Cole, Simon, and Cole [12] subsequently suggested that: "Some fields, such as physics, have a norm that submitted articles should be published unless they are wrong. They prefer to make 'Type I' errors of accepting unimportant work rather than 'Type II' errors of rejecting potentially important work." This suggestion might also account, at least in part, for the popularity of the arXiv eprint archives [3].

Hargens [13] reviewed previous explanations of the variation in rejection rates, which he found to be focused on two possible sources: space shortages and variation in consensus. He regarded variation in consensus as the more important determinant of rejection rates. Interdisciplinary variation in scholarly consensus involves the extent to which scholars share conceptions of appropriate research problems, theoretical approaches, or research techniques. When scholars do not share such conceptions, "they tend to view each other's work as deficient and unworthy of publication" [13].

Scholarly consensus seems likely to continue to be an important variable in the evaluation of eprints, even when acceptance for inclusion on an eprint server only depends on a favorable decision by the editorial staff of the server. Cole [14] has pointed out that: "Even at the research frontier ... minimal levels of consensus are a necessary condition for the accumulation of knowledge." Hargens [15] suggested that: "Perhaps a future study should examine the probability that a published paper will provoke a critical comment as a possible measure of scholarly consensus." From this perspective, perhaps rapid online responses to an eprint might provide a very convenient basis for efforts to assess the extent of scholarly consensus about the topics addressed in the eprint.

The establishment of some form of trust might be regarded as a crucial aspect of scholarly consensus. As Eysenbach has noted, "manuscripts may first be 'published' on the Internet, but 'establishing trust' may be a separate process and may have many different faces" [16].

## One Proposed Reform: Online Peer Review

The current consensus seems to be that, although there are problems with peer review, it is unlikely to be abandoned [17], but may be opened up [10]. Ideally, peer review should be reformed in ways that encourage innovation without a sacrifice of quality control [18]. One way to reform peer review is to develop new ways to undertake it online.

A case study of a journal that appears only in electronic form, and uses only online review, is provided by the Journal of Interactive Media in Education (JIME) [19]. JIME uses a three-stage review process. In the first stage, an article submitted (electronically) by its author(s) is assigned to three reviewers selected by the editor. The reviewers' comments, and the authors' responses, are posted on a private website, accessible only to the editors, reviewers, and authors for each submission.

In the second stage, revised articles that have been approved by the editors are posted, and identified as preprints, at the publicly-accessible JIME website [19]. Reviewers, readers, and editors (all of whom are publicly identified) may post comments. For example, editors may post summaries of comments, if the comments about a particular article become numerous.

In the third stage, the authors prepare a final version, which takes into account the comments that have been received, and submit it for final publication in the archives of the journal.

This process might be regarded as a "gold standard" for online peer review. However, it takes time, and requires a lot of effort by all of those who are involved. It seems unlikely to be practical unless the number of articles is quite small (JIME published 12 articles in 1998, and 2 in 1999 [19]).

Another example of an online review process is the one used by Sleep Research Online (SRO), where authors can monitor the progress of the review of their article using a private web page [20], but comments from readers (other than the selected review editors) are not sought.

Might the comments from self-selected readers be considered as a substitute for comments from referees selected by the editors? Bingham and colleagues [21] have addressed this question, and concluded that: "Postpublication review by readers on the internet is no substitute for commissioned prepublication review, but can provide editors with valuable input from individuals who would not otherwise be consulted." In the next section, a proposal about ClinMed NetPrints will be based on this conclusion.

## A Proposal about ClinMed NetPrints

In an editorial about the launch of ClinMed NetPrints [9], it was not clearly stated to what extent the editors of BMJ plan to take proactive steps to solicit the revision of NetPrints and their submission for conventional peer review. Unless otherwise negotiated, authors of preprints posted at the ClinMed NetPrints website retain copyright, and could submit revised versions to any journal willing to accept them for conventional peer review.

The editors of BMJ (and of other journals) might be well advised to consider the NetPrints posted at the ClinMed NetPrints website as equivalent to articles that have been submitted directly to their journal. After screening the NetPrints using their usual editorial screening criteria, they could decide to invite selected authors to submit their NetPrints (or revised versions of them) for conventional peer review.

Thus, a posted NetPrint would, in effect, have been submitted, simultaneously, to a (potentially) large number of relevant journals. Editors of different journals might soon discover that they are in competition with each other for the solicitation of NetPrints that they found to be interesting! Authors might then find that they must choose among journals, and decide to which one they would prefer to submit to first for conventional peer review.

Such a process should have advantages for authors, especially those at an early stage in their research careers. Authors of articles deemed to be of interest could quickly find an appropriate publisher. Competition among journals (and among authors) might be expected to enhance both the quality of manuscripts and the efficiency of the publication process. It seems much less likely that editors of well-established, high-impact journals would find such a proposal appealing. However, editors of newly-established journals might welcome an opportunity to rely on an existing large pool of preprints into which they could dip in order to solicit submissions, especially preprints that clearly provide an excellent fit with their journal's particular "niche." Because of the advantages of such a process for a rising generation of researchers, editors of journals that refuse to participate in such a sequential publication process might, as time passes, find that they have lost some reputation, and hence, some impact.

Might comments about preprints, received from readers, provide valuable critical appraisal prior to subsequent revision and submission for formal publication? In theory, the answer should be "yes." In practice, for the preprints posted at the ClinMed NetPrints site, only a very limited number of responses have been received. For example, a search of the website on July 31, 2000 revealed only two publicly-accessible responses to the 20 NetPrints posted between December 1999 and July 2000. It appears that, in the absence of an appropriate incentive (such as a request from a well-respected editor for a peer-review commentary), responses may not be frequent, unless the topic of the preprint is an especially controversial one.

Of course, public access to these NetPrints provides an opportunity for their authors to solicit, from respected colleagues, constructive criticisms via private messages, or via one or more of the many online discussion groups and forums. An example of such a forum is provided by the archives of the September 1998 American Scientist Forum, moderated by Stevan Harnad [22].

It should be noted that, no matter which journal publishes an article, it seems likely that it will, at some point in time, become publicly accessible in a major electronic archive. Examples are JSTOR [23], and PubMed Central [6].

## Conclusion: More Evaluative Studies Are Needed

The major proposal presented here is based on the view that eprint servers such as the ClinMed NetPrints website provide a novel opportunity for the establishment of what Peter A. Singer has called " free market in knowledge" [24].

Preprints archived at the server could be regarded as having been submitted, simultaneously, to all interested and relevant journals [24], a model for publishing similar to Gunther Eysenbach's "paper auction" model, which suggests that in the future researchers will not submit their papers to journals, but first to preprint servers for discussion and peer-review, and journal editors and publishers pick and bid for the best papers they want to publish in their journal - the best journals would be able to pay the highest prices for the best papers, and the number of bidders or the sum bid for each paper determines its value [25]. This process has obvious advantages for authors, and may benefit scientific publishing in general. For example, the editors and publishers who adapt best to such a "free market" may be those able to demonstrate most clearly that they provide added value, via their editorial and peer-review processes, to the published articles (in comparison with the initial preprints).

Evaluative studies of eprints are needed. For example, might articles published initially as preprints, and subsequently revised on the basis of comments received (publicly or privately) from readers, be of higher quality than articles submitted directly to a journal?

When making such a comparison, what criteria should be used to evaluate the quality of articles? As noted above, most studies have "used the quality of the review as an outcome measure rather than the quality of the paper" [10]. This important issue will not be addressed further here, except to make two points. The first is that it would be helpful to researchers interested in the evaluation of eprints if every eprint archive included a (preferably, standardized, and publicly-accessible) set of statistics on usage. Such statistics might include data about the relative popularity of individual eprints, using measures such as the number of times a particular preprint is visited, the number of times it is downloaded, and the median duration of visits to it. For example, a collection of electronic theses and dissertations (ETDs) currently provides statistics about the ten most accessed ETDs [26]. The usefulness of such statistics as possible indicators of quality needs to be assessed, in comparison with more conventional criteria (see, for example, [27-29]). The second point about measures is to reiterate Tukey's warning: "when the right thing can only be measured poorly, it tends to cause the wrong thing to be measured, only because it can be measured well" [30].

## References

[1] Clinical Medicine NetPrints.

[2] The Lancet Electronic Research Archive.

[3] The arXiv Archives.

[4] The Open Archives Initiative.

[5] Ginsparg P Winners and losers in the global research village.

[6] PubMed Central.

[7] The Lancet's response.

[8] The New England Journal of Medicine's response.

[9] Delamothe T, Smith R, Keller M A, Sack J, Witscher B (1999). Netprints: the next phase in the evolution of biomedical publishing. BMJ.

[10] Smith R (1997). Peer review: reform or revolution?. BMJ.

[11] Zuckerman HA, Merton RK (1971). Patterns of evaluation in science: Institutionalization, structure and functions of the referee system. Minerva.

[12] Cole S, Simon G, Cole JR (1988). Do journal rejection rates index consensus. Am Sociol Rev.

[13] Hargens LL (1988). Scholarly consensus and journal rejection rates. Am Sociol Rev.

[14] Cole S (1983). The hierarchy of the sciences. Am J Sociol.

[15] Hargens LL (1988). Further evidence on field differences in consensus from the NSF peer review studies. Am Sociol Rev.

[16] Eysenbach G (1999). Challenges and changing roles for medical journals in the cyberspace age: Electronic pre-prints and e-papers [editorial]. J Med Internet Res.

[17] Böttiger L E (1999). Printed medical journals - will they survive?. J Intern Med.

[18] Horrobin D F (1990). The philosophical basis of peer review and the suppression of innovation. JAMA.

[19] Journal of Interactive Media in Education (JIME).

[20] Sleep Research Online: editorial review.

[21] Bingham C M, Higgins G, Coleman R, Van Der Weyden M B (1998). The Medical Journal of Australia Internet peer-review study. Lancet.

[22] Archives of SEPTEMBER98-FORUM@LISTSERVER.SIGMAXI.ORG.

[23] JSTOR.

[24] Singer P A (2000). Medical journals are dead. Long live medical journals. CMAJ.

[25] Eysenbach G (2000). The impact of preprint servers and electronic publishing on biomedical research [editorial]. Curr Opin Immunol.

[26] Statistics on the Usage of the Virginia Tech Collection.

[27] Hernández-borges AA, Macías-cervi P, Gaspar-guardado MA, Al et (1999). Can examination of WWW usage statistics and other indirect quality indicators help to distinguish the relative quality of medical websites?. J Med Internet Res.

[28] HighWire Press Usage Statistics.

[29] ResearchIndex: The NECI Scientific Literature Digital Library.

[30] Tukey JW (1979). Methodology, and the statistician's responsibility for BOTH accuracy AND relevance. J Am Stat Assoc.

